# Stakeholder perspective on barrier to the implementation of Advance Care Planning in a traditionally paternalistic healthcare system

**DOI:** 10.1371/journal.pone.0242085

**Published:** 2020-11-10

**Authors:** Stellar Hiu, Alex Su, Samantha Ong, Daniel Poremski

**Affiliations:** 1 Health Intelligence Unit, Institute of Mental Health, Singapore, Singapore; 2 Medical Board, Institute of Mental Health, Singapore, Singapore; 3 Department of Nursing, Institute of Mental Health, Singapore, Singapore; Chinese Academy of Medical Sciences and Peking Union Medical College, CHINA

## Abstract

**Background:**

Advance psychiatric agreements could guide medical teams in providing care consistent with the incapacitated service user’s wishes. However, these types of agreements are rarely completed in Asian settings. What challenges can a traditionally paternalistic healthcare system expect to encounter when attempting to implement psychiatric advance directives?

**Methods:**

We answered this research question by exploring the cultural, administrative and logistical challenges that might impede the implementation of the system supporting the service. We interviewed key stakeholders, 28 service users and 22 service providers, to seek their views and interests in the implementation of directives. We structured our analyses along a literature-review-based framework designed to guide further implementation studies, proposed by Nicaise and colleagues (2013). Accordingly, we divided our inductively generated themes into four longitudinal categories: pre-development stage, development stage, implementation stage, post-implementation stage.

**Results:**

Overall, the findings indicated that many service users and service providers are interested in advance care planning. They believed that foreseeable challenges could be overcome with appropriate measures. However, the multiple challenges of implementation led some service providers to be ambivalent about their implementation and led service users to dismiss them. Specifically, factors related to the local culture in Singapore necessitated adjustments to the content and structure of the directives. These include language barriers in a multicultural society, conflicting wishes in a collectivist society, taboos for speaking about undesirable outcomes in a traditionalist society, and time limitations in a fast-paced society.

**Conclusion:**

While culture-specific changes may be required to enable service users in a small Asian country to employ existing advance psychiatric agreement approaches, service providers and service users see their benefits. However, service providers must be mindful not to assume that service users are willing to defer every decision to their physician.

## Background

In the event of a psychiatric crisis (e.g., psychotic episodes), advance psychiatric agreements could guide medical teams in providing care consistent with the incapacitated service user’s wishes. This involves a series of voluntary and non-legally binding discussions between service providers and service users. Such discussions must be held when the service user is competent, usually when their illness is in remission, and able to provide input to the types of treatment they wish to receive should they become incapacitated again [1, 2]. It may entail documenting their preferences for medications, treatment procedures, hospital admissions and emergency contacts, to name a few [3–7]. Advance psychiatric agreements are designed to reflect the values and long-standing opinions that service users endorse in their lives [8].

Research has shown that the timely completion of such advance psychiatric agreements, or advance care plans (ACP) could provide many advantages. Service quality, for instance, may improve [9]. There could be a reduction in the need for involuntary admission and treatment [10]. Service users may also perceive a more positive experience of care [11–13]. Amidst these promising benefits, however, evidence suggests that the completion of an ACP remained low [14–17]. Reasons include the administrative burdens of care plans [18], the uncertainty of service user’s mental capacity to inform their care plans [19, 20], and cultural taboos for having to hypothetically speak about matters pertaining to one’s deteriorating condition [21], a taboo which is particularly present in the Asian cultures that make up Singapore’s population. Moreover, the service user’s documented wishes in the ACP may be difficult to adhere due to a multitude of reasons, ranging from the practical to the legal [2, 22]. For example, those under mandatory treatment orders, as provided by civil commitment orders, will likely not be able to choose the conditions of their next treatment [23]. Practically, a physician may not be able to follow wishes if the documented intervention is insufficient to bring about clinically significant improvement in the individual’s condition. Clinicians thus do retain some leeway to override previously documented wishes. This may in turn fail to provide any significant improvement to the therapeutic alliance as research had suggested [24], or at worse, jeopardize it, thereby calling into question the merits of completing an ACP.

Aside from the theoretical gaps, there are also foreseeable cultural challenges for setting up ACPs. Numerous Asian countries have been slow to adopt legislation that supports ACP practices [25]. This may result from the perception that people with mental illness fall under the legislation protecting people with disabilities [25] or may result from the vestiges of colonial legislation that have only recently been shed [26]. In this way, the interplay of politics and culture play an important role, especially when updating practices to move beyond colonial legislation becomes politically important. In Singapore, for instance, locals have lived by the same political system since it gained independence in 1965. Largely attributed to the founding father Lee Kuan Yew, early social policies were so far-reaching they continue to have an impact today. The policies have also contributed to the rapid developments in Singapore, propelling it from ‘third world to first’ [27]. Despite having received much criticisms for the same authoritarian outcomes in every general election, or in certain parts of their leadership styles, Singaporeans largely trust the paternalistic government, as surveys regularly indicate [28]. In Singapore, this paternalistic culture permeates local organisations, especially in the healthcare sector. “Medical paternalism serves the patient best [29].” The principle of beneficence from the doctor’s perspective often drives the dynamics of healthcare provisions in Singapore [30]. With years of education and training, doctors are seen to be better informed to treat a service user’s medical condition. Because paternalism permeates, the authority of physician is not usually disputed and service users, in turn, express high levels of trust in letting the physicians direct their treatment [31]. Additionally, the numerous cultures that make up the Singapore mosaic [32] each have different sets of traditions and beliefs that impact their decisions for medical treatment in general, despite the fact that the country seeks to merge local and Western practices [33].

Given that there are many divided opinions on the practicality of ACPs [2, 4, 7], it is difficult to determine if setting up an advance psychiatric agreement programme would indeed improve mental health services. Existing work have been quite keen in highlighting the potential benefits that advance statements (or directives) have on the processes of care, and this is typically evaluated through randomised controlled trials [34, 35]. While this methodology is appropriate in measuring the effectiveness of the intervention, it provides little information to the implementation and delivery of an intervention as complex as advance agreements [24, 36]. Since implementing complex interventions requires context-specific solutions, questions remain as a result of these gaps, especially in what concerns Asian contexts [25]. Putting in place a system for ACP without an adequate understanding of the potential barriers could compromise the exercise [7, 22].

### Aims

Given that ACP have shown promise in other contexts, but might be challenging to transplant into an Asian context as conceived of in the Western context, there are two aims to this paper: 1) To determine service user and service provider interest in ACP, and 2) To determine the logistical and administrative challenges that might impede the effective implementation of a system for ACP with consideration for psychiatric agreements.

## Methods

All procedures performed in studies involving human participants were in accordance with the ethical standards of the institutional and national research committee and with the 1964 Helsinki declaration and its later amendments. Ethics approval was obtained from the institute’s ethics review committee (Clinical Review Committee reference #613–2018), as well as the national ethics committee (National Health Group Domain Specific Review Board, Domain F1 #2018/00247). Written informed consent was obtained from all individual participants included in the study prior to their participation. Service user participants were screened by their treating physician for fitness to participate.

This qualitative project sought to obtain the perspectives of service users and service providers to determine how each group viewed the use of ACP. It was nested under a larger qualitative project that sought to obtain the participants’ perspectives on treatment choices and which choices they would like documented in their ACP. Service user participants were individuals diagnosed with psychosis-related disorders, and service provider participants worked with service users with psychosis-related disorders. Digitally recorded semi-structured interviews were conducted and transcribed verbatim. Transcripts were first inductively coded to let themes emerge, and then deductively coded to fit the findings into an existing theoretical framework developed by Nicaise and colleagues [24]. Logistical and administrative barriers in implementing ACP are described below. Barrier-specific recommendations are presented in the discussion section. We followed the Consolidated Criteria for Reporting Qualitative Research, an atheoretical guide, to structure our manuscript [37].

### Setting

Singapore is a small equatorial island nation that is composed of predominantly Chinese, Malay, and Indian people [38]. Accordingly, the country observes the religious customs of each group, Confucian, Taoist, Muslim and Hindu [32]. This cultural plurality has led to an eclectic mix of various cultural and religious beliefs, all coexisting with Western ideals of free market economic systems [33]. This mosaic traces its roots to a British colonial past [27, 32]. While the predominant medical model resembles Western systems, picking from American and British systems [39], other models of care exist, including traditional Chinese medicine, Hindu and Muslim spiritualism [40–42].

Concerning psychiatric care in general, the host institute where the data was collected is the largest source of tertiary psychiatric care in Singapore. It has a capacity of approximately 1800 beds divided between long-stay (6 month or more) and acute-stay wards. It has specialized wards for first episode psychosis, mood disorders, substance use disorders, rehabilitation, as well as geriatric and palliative care wards. Its occupancy rate fluctuates between 85% and 95%. It hosts a wide variety of outpatient services including specialist follow up, occupational therapy, and psychological services. It has the only emergency service dedicated exclusively to the treatment of psychiatric emergencies in the city, which serves approximately 16,000 cases a year [43]. A special interest group dedicated to advance care and psychiatric directives was formed in 2014. Interested staff met to share experiences and discuss the implementation of such practices. As part of this interest group, workshops were held yearly to allow staff to gain experience with facilitating the completion of ACP.

### Theoretical framework

The goal of an ACP is to maximize the chances of recovery while minimizing unwanted interventions. Despite the intuitive appeal, multiple drawbacks have led opponents and proponents alike to underutilize ACP [44]. We pulled heavily on the theoy constructed by Naicaise and colleagues, who applied implementation theories to the development of ACP services. The theory postulates that each stage of development will be accompanied by a stage-specific set of obstacles that should be overcome in order to facilitate successful transition through the stages and ultimately successful implementation of services. While such a theory is not necessarily content dependent (the development of any new service may fit into a staged model of implementation) listing the barriers sequentially in each of these stages allows for a systematic overview of processes. Expanding on the three stages developed by Nicaise and colleagues [24], the present paper describes four stages where barriers occur: pre-development stage, development stage, implementation stage, and post-implementation stage. The pre-development stage is the additional stage proposed in this study, as inspired by previous researchers [45, 46]. It describes intangible barriers, such as misconceptions or conflicting perceptions of ACP, that exist even before the service is created. The development stage describes the process of developing the system, such as the content of the document and selecting the right service user for the service. The implementation stage describes barriers such as dealing with conflicting wishes for the document; the final stage of post-implementation describes system-level barriers, such as accessing the information in the document, and legal matters concerning the overriding of the document under the mental health act.

Overall, a system for using ACP is notably complex as it embodies principles and care concepts meant to be used in various ways across stakeholders in a typically disjointed healthcare system [22]. The present paper thus draw on the above features to determine the challenges of implementing ACP in a different context to which it has not been generalized.

### Participant sampling and recruitment

In total, we recruited 28 service users with psychotic-related disorders and 22 service providers (i.e., staff) who worked with them. We chose to focus on service users with psychosis-related disorders because in comparison to those with milder forms of mental disorders, which are largely more accepted and better funded, people with more chronic and persistent forms of mental disorders are often neglected in planning and budgeting [47]. Additionally, people with psychotic-related disorders alternate between periods of competence and incompetence regularly [47].

People with psychosis-related disorders, who were receiving inpatient and outpatient services were eligible if they were between the age of 21 and 65. Those with comorbid diagnoses of intellectual disability or dementia-related diagnoses were excluded, as were those who did not understand the research procedures. Inpatients were recruited only from the hospital’s rehabilitation ward, as this pool of service users was preparing for discharge and were well. Outpatients were recruited by means of referral from their attending physician.

We began sampling service providers by interviewing the handful of them who had been trained to conduct ACPs in general medicine as part of the institute’s ACP interest group. Those who were interested in this interview responded to an email invitation. Subsequent recruitment followed a snowballing technique independent of people’s participation in the ACP interest group [48], where previous participants recommended who else to interview based on the information discussed during their interview. This was done to acquire a range of perspectives from the respective stakeholders yet maintain a focus on those with relevant knowledge of advance care.

### Data collection

Digitally recorded semi-structured interviews were conducted between June 2018 and April 2019. We chose to stagger the interviews with the two groups of participants, beginning with staff. A recruitment overlap allowed us to use theory emerging from the service user groups to query staff perspectives. It also allowed us to develop an understanding of the issues on which to base our service user interview guide and sampling approach.

We developed two general interview guides based on existing literature done in other fields of care where ACPs are relevant [49–52]. Additional questions were posed to fill in the gaps as identified in the theoretical framework used for this study. For instance, defining the right moment to raise the topic about an ACP to the service user was found to be a common barrier across various studies [21, 53], but not in Nicaise and colleagues (2013). These guides thus take into account a holistic overview of processes that would facilitate the effective implementation of ACP (see S1 File).

For staff, we queried their understanding of ACP, the challenges they expected, and their roles and responsibilities. For service users we queried their illness experiences, the periods in their life when they lost capacity, the decisions made for them and the decisions they would like to make for themselves. After discussing these elements, we introduced the ACP concept and explored their interest. We did not query the ACP immediately because service users may lack familiarity with them. Finally, we queried whether certain limitations of ACP might compromise their therapeutic alliance with their service providers, notably the condition by which physicians could over-ride the ACP.

Staff interviews were conducted by the senior author, lasted an average 48 minutes (SD 12), and were conducted in the staff’s office. The first author and senior author conducted the service user interviews predominantly in pairs. These lasted an average 44 minutes (SD 12) and were conducted on the IMH campus in outpatient offices or in inpatient offices. These environments would have been familiar to service users.

We placed our questions in an order which reversed our aims, as we knew that service users may not be able to immediately answer questions about their preferences for ACP and because we wanted service provider participants to reflect on their opinions, both positive and negative, before providing a decision about ACP.

### Data analysis

Interviews were transcribed by the research team shortly after each interview to allow the team to reflect on the interview style. Nvivo 11 was used to facilitate the coding.

It is important to note that the order of the aims was reversed in the analyses, commencing first with the analysis of the content related to the processes required to implement advance care. We then proceeded to the analysis of the content related to participant interest in ACP. This is because the order of the aims reflects certain epistemological priorities of the local context; and the order of the analyses follow the flow of the interviews, ordered, as noted above, specifically to facilitate the co-creation of knowledge with the participants.

To determine which administrative processes would need to be implemented to facilitate the use of ACP, we used a content analysis [54] to analyse the data. The first author and senior author searched for and coded barriers and facilitators to the implementation of these services. The coded text (unit of analysis) was set to be wide enough to contain the context of the quote, but narrow enough to avoid missing the key elements of the unit. This was approximately 4–6 sentences, as agreed upon by the team. Descriptions of each code were created by the team and stored in the software. When adjustments were made to the use or meaning of a code, the old code was tagged with a prefix to indicate that it was the older version, and a new version with the modified meaning was created. Codes were discussed and various elements that emerged from our data were then organized into themes along the theoretical framework noted above. We did not restrict the coding to content related to the theoretical framework, and used an inductive approach to ensure that blind spots were not omitted.

We considered participants’ interest in ACP as manifest content and categorized their opinions. We intended to split the participants between those who were in favour of ACP and those that were opposed by coding the section of the interview where we had asked participants to sum up their thoughts on the topic. However, as noted below, the data resisted binary coding and multiple categories emerged. For both areas of focus, we used primacy and intensity to assign importance to the content. An audit trail was kept by using Nvivo’s tracking function, which logs each activity by each user.

### Reflexivity

The team is composed of a qualitative method specialist trained in psychiatry, senior consultant psychiatrists, and a researcher trained in psychology. None of the team has first-hand experience with service use. The qualitative researcher has conducted several previous projects in the same setting, but is not of the same ethnicity as the local population. The other team members, including the first author who analysed and interpreted the data, are Singaporean. The ethnic difference between the lead interviewer and service users might have contributed to the information which the participants were willing to report. Conducting the interviews with service users in pairs mitigated this effect and allowed the team to pick up on culturally relevant elements during the interviews. The team believes that interviewers had good rapport with participants because participants spoke the majority of the time with minimal input from the interviewers.

The order in which we approached the collection of data may influence the results of our analyses. We are conscious that the way in which we co-created knowledge with our participants had an effect on our conclusions. Specifically, asking staff participants to list their impressions of ACP and its implementation before asking them to sum up their impressions in a recommendation may have led them to take stock of the multiple barriers to implementation prior to formulating a summative opinion. Therefore, their results may lean more toward a reduced interest in ACP because of the perceived barriers. Conversely, because we asked people to talk first about their general health seeking experiences and the difficult periods in their life when they had lost capacity before we introduced the topic of ACP, it is possible that service user participants had a more favourable view of ACP as it was offered as a potential solution to eliminate coercive practices in the future. We defend our choice of co-creating knowledge in this order and temper our conclusions accordingly.

## Findings

### Demographics

A total of 50 people participated in our qualitative interviews (28 service users, and 22 staff). Thirteen of the staff participants and 16 of the service user participants were women. Staff composition was as follows: 9 psychiatrists, 7 nurses, 3 medical social workers, 2 case managers and 1 pharmacist. Of the service providers, 14 were Chinese, six were Indian, and two were Pilipino. None was Malay. By virtue of the recent introduction of advance care and psychiatric directives to the institute, staff had relatively uniform experience with ACP. Not all had received specific training on facilitating ACP, but the majority had some involvement with the institute’s ACP interest group. Details of service user participants are given in Table 1.

**Table 1:** Service user participant demographics.

	Mean	SD
Age	39	11
Education	13	4
Comorbid medical conditions	4	3
	n = 28	%
Employment status		
Full time	9	32
Part- time	4	14
Volunteer or student	2	7
Unemployed	4	17
Special work programme	9	32
Ethnicity		
Chinese	15	
Malay	7	
Indian	5	
Other	1	
On public assistance	4	14
Receive Medifund*	14	50
Relationship status		
Divorced	5	18
Married	2	7
Single	21	75
Maintains Contact with parents		
Parents Deceased	4	14
No	2	7
Yes	22	79
Housing status		
Private home owned by parents/ family	10	36
Private home owned by participant/ spouse	5	18
Renting under participant/ spouse	1	4
Renting under parents name	1	4
Supported accommodation	11	39

* Medifund is healthcare-specific social assistance.

### Service user and service provider interest in advance care planning

The first aim of this study was to explore participant’s interest in using ACP. This was determined by getting participants to summarize their opinions about advance planning and its feasibility at the end of each interview. We originally planned to split the participants between those who were willing to embrace the principles of ACP, and those that were satisfied with the existing standard of care. Such a binary division was logical and would allow us to compare and contrast the two groups and determine which points we could leverage to bring about a greater acceptance of ACP, and thereby overcome the respective barriers.

Ambiguities arose, however, making a binary distinction between adopters and non-adopters difficult. Responses were therefore placed along a continuum between our two original poles as appeared logical to us: for service users, the categories were 1) strongly agree, 2) agree, 3) neutral, 4) disagree, 5) strongly disagree, and 6) did not contemplate ACP; for service providers, categories were 1) agree, 2) neutral, and 3) disagree. Service provider responses were more straightforward, probably because they were already familiar with the principles of ACP. The descriptions of each category and the results are presented in Tables 2 and 3. While these categories are our own classification of interest, they pull on common forms of interest grading to facilitate interpretation.

**Table 2:** Service user degrees of interest in ACP, and corresponding number of participants that expressed interest.

Service users		
**Strongly agree**	Strongly advocated the service with some having thought about ACP previously.	**7**
**Agree**	Tended to show some inclination towards the service (able to describe preferences for future treatments), but did not elaborate on their preference. Had previously made requests known to attending physicians	**5**
**Neutral**	Saw the benefits of the service but also identified many challenges	**1**
**Disagree**	Would rather rely on others to make decisions for them; did not see the need, or was unable to plan ahead of time	**9**
**Strongly disagree**	Strongly discouraged the service	**0**
**Did not contemplate ACP**	No opinion; did not immediately grasp the intention of ACP*; limited contribution to the discussion;, or raised to them but participant did not answer	**6**

* See Limitations.

**Table 3:** Service provider degrees of interest in ACP, and the corresponding number of participants that expressed interest.

Service providers		
**Agree**	Strongly advocated the service. Have identified various challenges but deemed that the challenges can be overcome. Benefits outweigh challenges.	**11**
**Neutral**	Saw the benefits of ACP but also deemed many challenges difficult to overcome	**9**
**Disagree**	Strongly discouraged the service. Thought that challenges outweigh the benefits.	**1**

For service users, the results show a mix of participants who wished to complete their care plan and those who were satisfied with their existing standard of care. One participant who was in favour of completing the ACP noted:

"*I think it is important that we do that. We still do advance care planning. At least we have safety plan. At least we have something to fall back on. Rather than not having the plan at all. Because not having a plan at all, just liberates everyone to make every decision they want to make on it. And you don’t really have a, a 1.10% of say. At least, when you do a plan, umm, you have already state what you wanted, and what you’re comfortable with, and whether the medical team eventually do carry it out, is depending on them and not on you anymore. That’s not something that you can control, but you already have state your demands, set your boundaries, I think that is enough. I mean, ya… you’ve done as much as you can to help yourself*." *[20013, service user]*

Service users who were satisfied with their existing standard of care expressed high dependency on others to make treatment decisions on their behalf:

*"I will leave it to the doctors lah… they’re the professionals… they should do their job lah…" [20028*, *service user]*

While it is difficult to explore the impact of age in the current study, it is notable that the younger participants spoke more of the various types of plans they had initiated to prepare for an eventual relapse, whether it was a crisis plan, an informal ACP or a registered directive. Older participants appeared to be more satisfied with deferring the issues to their physicians.

Unlike service users, service providers did not generally disagree with the use of ACP, but rather were neutral about their use or agreed that the service would be beneficial. While they saw the benefits, they saw many challenges that would make the implementation unfeasible.

For those who advocated for the implementation, one participant described:

*"ACP is good as it gives peace of mind to the service users and family knowing the decisions have been made. It is simpler to have the decision made prior to their need, rather than not have the information/decision and need it urgently." [Participant 25*, *professional]*

For those who were neutral about service, the multitude of barriers they identified were the cause of their apprehension. These barriers are organized along the ACP’s longitudinal development, and are discussed below.

### Logistical and administrative processes

The second aim of this study was to determine the logistical and administrative processes required to effectively implement a system for ACP. The analyses of the findings were structured in a way that pulls on the framework proposed by Nicaise and colleagues (2013). This allowed us to divide our inductively generated themes into four categories along the longitudinal development: pre-development stage, development stage, implementation stage, post-implementation stage. A summary of the barriers in each of these stages can be found in the Table 4.

**Table 4:** Organization of the barriers along the 4 stages of implementation.

Stages	Barriers
Pre-development stage	• Low awareness • Conflicting perceptions and misconceptions
Development stage	• Selecting the right service users for the agreement • Types of contents to include in the document • Person-in-charge of initiating the document
Implementation stage	• Language barriers • Conflicting wishes for the document • Cultural taboos about discussing undesired outcomes • Service provider attitudes
Post-implementation stage	• Ease of access • Complexities in cross-system collaboration and IT systems • Honouring and overriding wishes

### Pre-development stage

This stage forms a large bulk of this exploratory study as we sought to understand the views and perceptions of both service users and providers on the topic. Various limitations were identified, including **low awareness** and **conflicting perceptions** of ACP.

As Singapore has never had a centralized systematic system for using ACP in psychiatry, there was **limited awareness** on the process amongst staff and service users. Only a handful was aware because they had attended courses related to ACP. Others possessed some knowledge on end-of-life care, which is common in the medical setting, and were thus able to relate to ACP when it was addressed in reference to end-of-life care.

*“Because as of now, if it is not implemented at all, there isn't even the checking in on the preference, so the complaint is& nobody even knows that there is that piece of paper.” [Participant 17*, *professional]*

Some participants expressed **conflicting perceptions** to the goals of ACP with consideration for psychiatric agreements. For instance, instead of facilitating treatment decisions, documented wishes may obstruct treatment decisions when they are not aligned with the service user’s best interest.

*“It may facilitate, but on the other hand, let’s say. For example, a person with schizophrenia has done an ACP, and he or she does not want neural stimulation options. But perhaps when the person is unwell and admitted to hospital, that might be the only and best treatment option available… and so, his decision on the ACP will have to be overridden in such a situation… so then, so that doesn’t really help to facilitate treatment, but rather obstruct treatment, in that sense…” [Participant 35*, *professional]*

Additionally, even though the document advocates service user autonomy in healthcare provisions, many service users would rather rely on the service providers to make treatment decisions on their behalf.

*"yeah I think there’s no reason because I find my doctor is fine… I mean, whatever he does*, *he does for my best… " [20016, service user]*

"I’m also not really sure what to do because I’m not those decision-maker… so, I just let them do what they think is better, because I’m not really sure what’s… um… better for me. Ya… and they know more than me, so…" [20010, service user]

Some service providers also pointed out the high degree of paternalism in Singapore, which would act as a barrier to implementing ACP:

*“I think there would be barriers… and I think it might be particularly difficult to implement… in Singapore which is still largely quite paternalistic*, *I feel… as opposed to somewhere like the States… if I’m not mistaken, I think ACP is available in certain states within the US… not sure about Europe or UK… but in general, I think these countries do value service user’s autonomy quite a bit. And therefore I can imagine the actual implementation of it, might be a little bit different from what it’s going to be like if it’s implemented in Singapore.” [Participant 35, professional]*

This is especially relevant in the psychiatric context, where service providers may question the service user’s mental capacity and level of insight. Service providers believed that people with low levels of insight were unamenable to the completing an ACP.

*“… if they don’t believe they have an illness*, *how can they make a care plan to manage the illness… so, because for them, they’re normal. These delusions are real. For them it’s real. If they could be reassured, if they could be reasoned, then they wouldn’t be in the hospital, they wouldn’t be needing medication, because they’re fine. It’s just, you know… it’s just… so this is fixed, non-shakeable… if they say no no no… this is happening, why should I take the medicine. Then it’s going to be very difficult.” [Participant 1, professional]*

### Development stage

This stage involves understanding the types of preparation (or training, standardization) that would be required before rolling out the service to service users. It includes knowing **which service users to select**, **what to include in the document** and **who to initiate the service**.

Service providers found it challenging to imagine **what types of service user** would be suited to completing an ACP. Service users who were new to services were believed to possess insufficient knowledge about the various treatment alternatives to make informed decisions, and those who were too old were seen as too set within the “patient role” to accept the idea of informing their care.

*“… what kind service user is possible… too young*, *cannot… too old, cognitive wise unless family is involved and things like that… but they must be, to some level, stability… otherwise it’s not very helpful for them.” [Participant 41, professional]*

As we noted above, some service users who had good service use experiences were willing to defer to their treating physician, meaning that service user who were in a good working alliance may be the easiest to talk to, but also the least likely to see the need for an ACP. Whereas those with negative experiences may be more reluctant to engage with services, but may be most willing to document an ACP.

Some **disparities in the contents of the document** could also be observed between the two groups of participants. In general, service users generated more themes in their preferences for the document than service providers–while service providers tended to focus only on treatment preferences, service users also expressed non-treatment preferences: *“And just by showing empathy*, *and being there for me*, *those are the things I’d want*.*” [20012*, *service user]* The challenge is to determine how concise the document ought to be–too much will result in information overload, too little will provide physicians with insufficient information.

Basing the discussion as a joint crisis care plan initiative, there were questions about **who could initiate** the document, and when. Some participants preferred that service users themselves, for their sheer interest, initiate the process. Others took on a more ambivalent stance that anyone with good knowledge of the process and of the service user could do it. Still others, were able to pinpoint the exact profession who should initiate the process, but suggestions varied, including doctors, nurses, or social workers.

### Implementation stage

The stage involves understanding the challenges that the respective stakeholders may face during the discussion (or negotiation) process of ACP. Various cultural elements unique to this study were addressed, including **language barriers**, **conflicting wishes for the document**, **cultural taboos**, and **service providers possessing the right attitudes** during the ACP’s discussion.

Because Singapore is a multi-racial and multilingual society, **language barriers** are bound to occur. This, unfortunately, was deemed to have a significant impact on the way message is communicated and understood:

*“most of my patients who are dialect-speaking*, *so I get an interpreter from the medical records office, because I want to be convinced that she completely understands in the language she’s comfortable with. So that’s another complexity you have to pitch it, it’s my problem, I don’t know Chinese.” [Participant 1, professional]*

Singapore was also developed on the basis of many Confucian principles. One of it is the idea of collectivism, where individual exists in the context of family, friends, and the wider society. In line with this, decisions, including documented wishes in their ACPs, are to be made collectively with one’s family members. The difficulty here lies on having to reconcile **differences in opinions** (or wishes) that are to be documented into the agreement, or when the medical team is actually attempting to realize the wishes during a crisis episode. *“When we were at training*, *we were told*, *that there were also incidents where the family quarrelled even though there is that piece of paper written up” [Participant 17*, *professional]*

Being a majority Chinese (76.2%) country, some entrenched beliefs and customs exist in the local culture. One of it is the abstinence of undesirable outcomes, such as death. Death is a huge **taboo** topic among many Chinese. Because some misconceptions arise about ACP being solely relevant to end-of-life statements, brief mentions about the document would incur thoughts of death. Chinese believe that discussing mortality would beget bad luck and bring the inevitable nearer than it may already be. Parents refrain from discussing the topic with their children in order to protect them; people abstain from the digit 4 as it resembles the word ‘death’ in Mandarin; and few people register as organ donors or write their own wills for fear of cursing themselves. In line with this topic, many negative reactions to the advance agreement proposal would be expected.

*“a lot of our patient they will say that ‘If I ever plan for my future… it is just like now advance care for directive’*, *a lot of people are not doing it, ‘I am well, I am still so young, I am still so strong. why plan for someone, is it taboo, are you cursing me,’ that kind of thing…” [Participant 5, professional]*

There were also concerns about having the **necessary attitudes** during the ACP discussion. This is in view that Singapore is a fast-paced society, and that the additional service would pose greater workload on the already time-restricted medical team. This would translate into a task that meets bureaucratic expediency, rather than to serve dynamic user centred needs.

*“I think it is the time taken… so it will take more time to engage*, *I need to set aside time, cos you don’t want to rush these things… but at the same time, we don’t want this to be a mindless paper exercise, where you just put in another piece of document, it gets filed somewhere and nobody really pays attention to it. And it doesn’t really mean anything… so I think if you do do that, it has to be done meaningfully… patients were engaged in the process, know what they’re giving, expressing their preferences for and about… and physicians take it seriously…[…] otherwise, it’s a waste of time…” [Participant 37, professional]*

If service providers held such an attitude, people worried the intention of the exercise would be lost.

### Post-implementation stage

This stage involves many system-level processes, where the documented wishes are stored into the system for later retrieval. Barriers concern the **ease of accessing** that information and **cross-system collaboration**. There were also many **concerns about reconciling the service user’s wishes** in situations where the Mental Health Act is called upon to enforce protective measures.

Many variations of ACP are already in place in Singapore, such as living will, the lasting power of attorney and do not resuscitate order. The addition of psychiatric agreements into the current work processes poses a challenge to physicians when it comes to **accessing the information** in the most convenient way possible.

*“if you’re going to systematize it*, *you could do it, but do it together I think. Otherwise it’s just two different systems in place. Already there’s so many different things, there’s the MCA, [mental capacity act] then there’s this ACP, then there’s AMD [advance medical directive], and so on… so, I kind of thought if you could kind of combine with the ACP for physical condition, I think that would be a nice kind of dovetailing, I think, to start off…” [Participant 31, professional]*

The sharing of information between organizations (or hospitals) also appeared to be a complex one as there may be a lack of **cross-system collaboration** compounded by IT systems. This fragmentation of existing service and limited resources make the implementation of ACP difficult to realize in practice.

*“I think it’s quite difficult… because even to access notes from the another hospital*, *it’s quite tricky, you need consent from the patient, you need to go through medical records office, MRO needs to process… and they need to get their own hospital data… guardian or custodian to approve to release of information… so it’s a very tedious process. […]. If you do want to have an electronic document that can be shared across institutions, particularly across clusters… and I think if… even if the patient were to carry their own document, oftentimes they lose the document and they seldom remember at all, or have it with them, particularly when they’re unwell, and need that document…” [Participant 37, professional]*

Should the need arise, service users may be subjected under the Mental Health Act to receive services against their will. People questioned how these situations could be **handled and the two forces reconciled**. The effort to conduct the ACP process may prove futile if it fails to take into account these implicit coercive elements of mental health services, as service users noted:

*“Because if I went through the trouble of making an emergency plan… then, then they didn’t adhere to it, then what would be point of making the emergency plan in the first place? Ya… I will be mad if they didn’t follow the treatment plan.” [20027*, *service user]*

## Discussion

The research question posited at the outset of this study was ‘What challenges can a traditionally paternalistic healthcare system expect to encounter when attempting to implement psychiatric advance directives?’ The study answered this by uncovering the administrative and logistical challenges that would surface when setting up the framework supporting the service. This information was obtained by interviewing service users with psychosis-related diagnoses and the service providers that care for them. Overall, the majority of service user and service provider participants believed that benefits outweighed the foreseeable challenges. They believed these challenges could be overcome with appropriate measures and preparation. This opinion was not universal and the multiple challenges service providers and users expected to encounter led some service providers to be ambivalent about its implementation and led some service users to dismiss them altogether. Unlike service providers, who might feel more obligated to agree with the merits of ACP, service users occasionally expressed clear disinterest in ACP. This disinterest surfaced because they believed that the role of treatment decision was that of the professional paid to provide care. These disinterested service users also believed that they were not the right ones to make those types of decisions, possibly because they have long assumed a passive patient role. This is somewhat supported by the slight but perceptible age difference between those who wished to complete ACP and those who did not. This appears in line with self-advocacy being higher in youth. Our distribution of interest in completing ACP amongst our service user participants indicates that service providers initiating ACP need to be prepared for varying responses and should have various work-flows that suit these varying interests, being fully prepared to complete the ACP for those who are enthusiastic, being prepared to revisit the issue with those who have building interest, and specific reminders not to dismiss completing ACP in those who have once refused it in the past. Various overlaps can be found between the current study and the existing framework used to capture barriers to the implementation of ACP. These include misconceptions and low awareness about ACP due to its novelty [2, 22], concerns about the level of details to include in the document [18], system-level barriers such as cross-system collaborations in accessing the information [18, 22], and issues of honouring challenging wishes [23]. Other theoretical overlaps include the paternalism philosophy [31], as service user participants could be seen to be inclined towards accepting the decisions made for them by their physicians without much questioning. Like Swanson and colleagues (2006), there were also apprehensions about service user’s mental capacity to complete their care plans, given that the nature of work in mental health settings often deals with people who may lack capacity during acute phases of illness [26]. Believing that by default people in acute phases of illness lack capacity may stem from the tendency to err on the side of caution, characteristic of the paternalism present in Singapore. While resorting to alternative medicine occurs frequently in Singapore [40], it did not surface as relevant to the discussion of ACP in our participants. This could be because our sample, with an average age of 39 were relatively young, whereas alternative medicines, especially traditional Chinese medicine are favoured by older Singaporeans [40, 41].

Aside from barriers that have already been established in literature, the present paper also highlighted various interesting findings. There are many factors about the local culture that will make the planning of psychiatric agreements in Singapore different from Western settings. Culture-specific barriers identified in the current paper include language barriers, conflicting wishes between stakeholders in a collectivist society [26, 30, 55, 56], taboos for speaking about undesirable outcomes that not only surrounds death [21] but also any event that might be seen as undesirable, and time limitations in a fast-paced society. Though somewhat unique to Singapore, some of these elements may be generalized to other cultures that practice similar policies or customs. Malaysia, Indonesia or India, for instance, are in many ways more multilingual than Singapore as they actively practice several dialects, on top of the four main languages that they have in common with Singapore [57]. China is also in many ways similar to Singapore, given that majority of Singaporeans are Chinese and have ancestral roots in them [58]. Some of the recommendations discussed below may thus be relevant beyond the local context.

### Practice implications

According to the literature, many of the barriers identified during the interviews could be partially addressed with the introduction of courses, trainings and workshops prior to the actual implementation of ACP with consideration for psychiatric agreements [22]. This is because a concept as new as this would foremost require greater awareness among the public about its existence [59]. Such wider training and advertising would not only serve to eradicate any misconceptions (i.e., taboo topics) about the idea of ACP, but to also raise greater intrinsic interest among service users who may have an idea of how they would like their future treatments to be. As some service provider participants in the current study had previously expressed a preference for service users to initiate the discussion themselves, such initiatives may likewise reduce the cognitive workload of the respective personnel documenting the service user’s wishes as the service users would already have known what they want to include in the document. On this note, however, strong evidence had been found for the service provider’s attitude that service users should initiate discussions being a barrier [60]. This may explain why some discussions end up being initiated tardily when decisions need to be made [60, 61]. Conflicting perceptions like this would have to be addressed in these trainings so as to equip service providers with the right skills and attitudes towards the discussion.

Trainings and courses could also be implemented to equip service providers with administrative skills needed in the documentation process. The contents of this document, for example, may vary because it is tailored to the individual and to the system in which they are treated. However, service providers should minimally cover some basic information, such as the service user’s preference for medication and hospitals, information on crisis and relapse symptoms, emergency contacts, and when appropriate, a surrogate-decision maker [24, 44]. Other additional information may include the specific types of services that the service users would like to undergo (e.g., electro convulsive therapy), some protective and de-escalating methods should the service user experience a psychotic episode, and instructions to inpatient staff [45]. These contents had generally garnered consensus from both participant groups during the interview, and which can also be found in existing literature [45, 62, 63]. Nonetheless, the findings from this study also suggest that service users have an interest in discussing non-clinical preferences, such as financial concerns compounded by career progress, dietary preferences, or sending memos to their employers for their leave of absence. The study by Henderson and colleagues [7] showed that the documentation of non-treatment preferences could significantly enhance service user autonomy. Service providers should thus be informed of the types of information that may add value to the document beyond its clinical content.

Service providers who have time limitations might prioritize work agendas such as risk assessments, over other long-term recovery goals such as ACP. This lack of alignment of care planning activities with the everyday life of service users may result in its redundancy or obsolescence. In such situations the service risks becoming a checkbox exercise that meets organizational goals at the expense of delivering the primary stated purpose of care planning. In order to reorient care plans to the everyday lives of service users, other professionals like peer support specialists may be engaged to complement the ACP service in order to take care planning in a different, more user-focused direction. This would steer the objective of the care plan away from organizational constraints, as well as the paternalistic and clinical norms of surveillance and control associated with statutory services [64, 65].

Some physicians may forgo routine queries of interest in ACP, because they mistakenly believe that their service users would defer to the physicians. This risks potentially missing opportunities to learn about their service users’ wishes. Documenting wishes, however, may serve the physician in situations where conflicting wishes within the family emerge in the absence of a formal document. This emerged in managing end of life care decisions [30].

In light of Singapore’s Mental Capacity Act (MCA), treatment decisions are to be guided based on the service user’s best interest; in the event that service users are deemed completely incapable of completing the document, the relatives of the service users are to be consulted as part in decision-making process under the MCA. A review by Menon (2013) provides a comprehensive overview of dealing with these difficult choices, which have not been covered here. Additionally, when the MCA is required, service user’s wishes may be overridden. In this regard, two measures may be considered in order to minimize the negative impact on the therapeutic alliance. First, users should be informed about the possible scenarios that might lead to their documented wishes being overridden. Second, users should be debriefed and informed about any overridden wishes in a structured and documented manner following any applications of care under the MCA. The intentions associated with the overriding of ACP should be clearly presented to the individual, and service providers might benefit from confirming that the rationale is understood. Given that ACP documents are expected to be reviewed following changes in the person’s experiences (such as following a psychiatric episode, or new experiences with treatment), debriefing provides the opportunity for service users and service providers to refine the document. Accordingly, clear communication serves to update the ACP as part of routine best practice.

## Limitations

Our results may not generalize to other populations and research is needed to extend our findings to different service user populations as we restricted our sample to service users with a psychosis-related diagnosis. Additionally, the system on which we focused is still planning its advance care strategy. While this allows us to provide timely input to service implementation, it does mean that many discussions had to work in the hypothetical rather than the concrete. Similarly, because the services are still in the early stage of development, administrators, who might eventually be involved in the daily operation of services, were not interviewed. We chose rather to focus on service providers who had existing knowledge of ACP. Concerning our sample of service users, it is important to note that the capacity to participate in research and the ability to communicate wishes for care did not invariably mean that the service user participants grasped the abstract idea of advance care planning. As such, we were very clear in our classification of service user interest to avoid categorizing those that did not consider ACP (last category in Table 2) by equating their interest in having physicians follow their treatment wishes (something readily understood) with their wishes to speak with someone about their future medical treatment to record their wishes in an ACP (something slightly abstract for some service users). Those that did not grasp the concept of ACP or did not answer are grouped in the last category of Table 2. We might have increased the number of people with a clear opinion about ACP by sampling service users with a clear opinion of the process, but we preferred to sample a wider range of service users to gather information on their treatment wishes first, an important point of the larger project under which this one was nested.

## Conclusion

Professionals and service users are interested in the potential contribution to care that could be made if ACP is implemented successfully. However, they perceive multiple barriers that in turn dampen their enthusiasm for completing ACP. These barriers can be divided along a development continuum and can be expected to surface at different staged in the implementation and use of ACP initiatives. It is important that policymakers and organizations anticipate the emergence of such barriers and consider which other culturally specific obstacles might arise over the course of implementation and initiation.

## Supporting information

S1 File Full interview guides used for service user and service provider interviews. (DOCX)

## References

[1] Brock D. W, A proposal for the use of advance directives in the treatment of incompetent mentally ill persons. Bioethics, 1993 7: p. 47–56. 10.1111/j.1467-8519.1993.tb00291.x 11651538

[2] Savulescu J. and Dickenson D., The time frame of preferences, dispositions and the validity of advance directives for the mentally ill. Philosophy, Psychiatry & Psychology, 1998 5: p. 225–46.

[3] Hoge S., The patient self-determination act and psychiatric care. Bulletin of the American Academy of Psychiatry and the Law, 1994 22: p. 577–586. 7718930

[4] Srebnik D. and LaFond J., Advance directives for mental health treatment. Psychiatric Services, 1999 50: p. 919–925. 10.1176/ps.50.7.919 10402612

[5] Swanson J., et al, Psychiatric advance directives: an alternative to coercive treatment? Psychiatry, 2000 63(2): p. 160–72. 10.1080/00332747.2000.11024908 10965546

[6] Wilder C.M., et al, Medication preferences and adherence among individuals with severe mental illness and psychiatric advance directives. Psychiatr Serv, 2010 61(4): p. 380–5. 10.1176/ps.2010.61.4.380 20360277 PMC3676902

[7] Henderson C., et al, How should we implement psychiatric advance directives? Views of consumers, caregivers, mental health providers and researchers. Administration and Policy in Mental Health and Mental Health Services Research, 2010 37(6): p. 447–458. 10.1007/s10488-010-0264-5 20099077

[8] Widdershoven G. and Berghmans R., Adance directives in psychiatric care: a narrative approach. Journal of Medical Ethics, 2001 27: p. 92–97. 10.1136/jme.27.2.92 11314165 PMC1733370

[9] Richards D., A, et al, Developing a UK protocol for collaborative care: a qualitative study. Gen Hosp Psychiatry, 2006 28(4): p. 296–305. 10.1016/j.genhosppsych.2006.03.005 16814628

[10] Campbell L., A and S. Kisely R, Advance treatment directives for people with severe mental illness. Cochrane database of system reviews, 2009 21(1): p. CD005963 10.1002/14651858.CD005963.pub2 19160260 PMC4161493

[11] Bee P., et al, A systematic synthesis of barriers and facilitators to user-led care planning. Br J Psychiatry, 2015 207: p. 104–114. 10.1192/bjp.bp.114.152447 26243762 PMC4523927

[12] Crawford M., J, et al, User involvement in the planning and delivery of mental health services; a cross-sectional survey of service users and providers. Acta Psychiatr Scand, 2003 107: p. 410–414. 10.1034/j.1600-0447.2003.00049.x 12752016

[13] Thornicroft G. and Tansella M., Growing recognition of the importance of service user involvement in mental health service planning and evaluation. Epidemiol Psichiatr Soc, 2005 14: p. 1–3. 10.1017/s1121189x00001858 15792287

[14] Ramsaroop S., D, M. Reid C, and R. Adelman D, Completing an advance directive in the primary care setting: What do we need for success? J Am Geriatr Soc, 2007 55: p. 277–83. 10.1111/j.1532-5415.2007.01065.x 17302667

[15] Tung E, E. and North F., Advance care planning in the primary care setting: A comparison of attending staff and resident barriers. Am J Hosp Palliat Care, 2009 26: p. 456–63. 10.1177/1049909109341871 19648573

[16] Cree L., et al, Carer's experiences of involvement in care planning: a qualitative exploration of the facilitators and barriers to engagement with mental health services. BMC Psychiatry, 2015 15: p. 208 10.1186/s12888-015-0590-y 26319602 PMC4553006

[17] Grundy A., et al, Bringing meaning to user involvement in mental health care planning: a qualitative exploration of service user perspectives. J Psychiatr Ment Health Nurs, 2016 23(1): p. 12–21. 10.1111/jpm.12275 26634415

[18] Simpson A., et al, Recovery-focused care planning and coordination in England and Wales: a cross national mixed methods comparative case study. BMC Psychiatry, 2016 16: p. 147 10.1186/s12888-016-0858-x 27184888 PMC4868048

[19] Swanson J., W, et al, Psychiatric advance directives among public mental health consumers in five U.S. cities: prevalence, demand and correlates. Journal of the American Academy of Psychiatry and the Law, 2006b. 34: p. 43–56. 16585234

[20] Ghooi R.B., Dhru K., and Jaywant S., The urgent need for advance directives in India. Indian journal of medical ethics, 2016 1(4): p. 242–249. 10.20529/IJME.2016.069 27731296

[21] Barnes K., et al, Acceptability of an advance care planning interview schedule: a focus group study. Palliat Med, 2007 21: p. 23–8. 10.1177/0269216306073638 17169956

[22] Kemp K., Zelle H., and R. Bonnie J, Embedding advance directives in routine care for persons iwth serious mental illness: the challenge of implementation. Psychiatric Services, 2015 66: p. 10–14. 10.1176/appi.ps.201400276 25554232

[23] Perkins H., S, Controlling death: the false promise of advance directives. Ann Intern Med, 2007 147: p. 51–7. 10.7326/0003-4819-147-1-200707030-00008 17606961

[24] Nicaise P., Lorant V., and Dubois V., Psychiatric Advance Directives as a complex and multistage intervention: a realist systematic review. Health & Social Care in the Community, 2013 21(1): p. 1–14. 10.1111/j.1365-2524.2012.01062.x 22452515

[25] Poremski D., et al, Psychiatric Advance Directives and their relevance to improving psychiatric care in Asian countries. Asia-Pacific Psychiatry 2019: p. e12374. 31872576 10.1111/appy.12374 PMC7027531

[26] Ratnam A., et al, Psychiatric Advance Directives in India: What will the future hold? Asian J Psychiatr, 2015 16: p. 36–40. 10.1016/j.ajp.2015.06.004 26168765

[27] Lee K., Y, From third world to first: the Singapore story, 1965–2000: Singapore and the Asian economic boom 2000, New York: HarperCollins Publishers.

[28] *Singaporeans' trust up in Government and media: Survey*. 2019 March 18.

[29] Lim L., S, Medical paternalism serves the patient best. Singapore Med J, 2002 43(3): p. 143–147. 12005341

[30] Krishna L.R., Best interests determination within the Singapore context. Nursing Ethics, 2012 19(6): p. 787–799. 10.1177/0969733011433316 22547489

[31] Poremski D., et al, The impact of stakeholder preferences on service user adherence to treatments for schizophrenia and metabolic comorbidities. PLoS One, 2016 11(11): p. e0166171 10.1371/journal.pone.0166171 27851771 PMC5112999

[32] Matondang S.A., Multicultural identity of Singapore as ex-British settlement and new Asian global city. International Journal of Humanities and Social Development Research, 2017 1(1): p. 49–61.

[33] Ang I. and Stratton J., The Singapore way of multiculturalism: Western concepts/Asian cultures. Sojourn: Journal of Social Issues in Southeast Asia, 2018 33(S): p. S61–S86.

[34] Ruchlewska A., et al, The effects of crisis plans for patients with psychotic and bipolar disorders: a randomised controlled trial. BMC Psychiatry, 2009 9: p. 41 10.1186/1471-244X-9-41 19589145 PMC2716324

[35] Papageorgiou A., et al, Advance directives for patients compulsorily admitetd to hospital with serious mental illness. British Journal of Psychiatry, 2002 181: p. 513–519. 10.1192/bjp.181.6.513 12456522

[36] Thomas P., How should advance statements be implemented. British Journal Psychiatry, 2003 182(6): p. 548–549. 10.1192/bjp.182.6.548-a 12777348

[37] Tong A., Sainsbury P., and Craig J., Consolidated criteria for reporting qualitative research (COREQ): a 32-item checklist for interviews and focus groups. International Journal for Quality in Health Care, 2007 19(6): p. 349–357. 10.1093/intqhc/mzm042 17872937

[38] *Population White Paper: A Sustainable Population for a Dynamic Singapore*. 2013, National Population and Talent Division: Singapore.

[39] Poremski D., et al, Selection of New Psychiatry Residents within a National Program: A Qualitative Study of Faculty Perspectives on Competencies and Attributes. Academic Psychiatry 2020 44(5): p. 545–553. 10.1007/s40596-020-01282-1 32705571

[40] Ng T.P., Tan C., and Kua E., The use of Chinese herbal medicines and their correlates in Chinese older adults: the Singapore Chinese Longitudinal Aging Study. Age and ageing, 2004 33(2): p. 135–142. 10.1093/ageing/afh016 14960428

[41] Feng L., et al, Use of complementary and alternative medicines and mental disorders in community-living Asian older adults. Archives of gerontology and geriatrics, 2010 50(3): p. 243 10.1016/j.archger.2009.04.013 20238433

[42] Lee G., et al, Complementary and alternative medicine use in patients with chronic diseases in primary care is associated with perceived quality of care and cultural beliefs. Family practice, 2004 21(6): p. 654–660. 10.1093/fampra/cmh613 15531625

[43] Poremski D., et al, Lost Keys: Understanding Service Providers' Impressions of Frequent Visitors to Psychiatric Emergency Services in Singapore. Psychiatric services, 2016 68(4): p. 390–395. 10.1176/appi.ps.201600165 27842469

[44] Swanson J.W., et al, Facilitative psychiatric advance directives: a randomized trial of an intervention to foster advance treatment planning among persons with severe mental illness. American Journal of Psychiatry, 2006 163(11): p. 1943–1951. 10.1176/ajp.2006.163.11.1943 17074946 PMC3747558

[45] Elbogen E., et al, Clinical decision making and views about psychiatric advance directives. Psychiatric Services, 2006 57(3): p. 350–355. 10.1176/appi.ps.57.3.350 16524992

[46] Wilder C., M, et al, A survey of stakeholders knowledge, experience, and opinions of advance directives for mental health in Virginia. Administration and Policy in Mental Health and Mental Health Services Research, 2013 40(3): p. 232–239. 10.1007/s10488-011-0401-9 22240937

[47] Ito H., Setoya Y., and Suzuki Y., Lessons learned in developing community mental health care in East and South East Asia. World Psychiatry, 2012 11: p. 186–190. 10.1002/j.2051-5545.2012.tb00129.x 23024679 PMC3449347

[48] Trotter R.T. II, Qualitative research sample design and sample size: Resolving and unresolved issues and inferential imperatives. Preventive medicine, 2012 55(5): p. 398–400. 10.1016/j.ypmed.2012.07.003 22800684

[49] Perez M., V, Macchi M., J, and Agranatti A., F, Advance directives in the context of end-of-life palliative care. Curr Opin Palliat Care, 2013 7(4): p. 406–10. 10.1097/SPC.0000000000000007 24152977

[50] Carr D. and Luth E., A, Advance care planning: Contemporary issues and future directions. Innovation in Aging, 2017 1(1): p. 1–10. 10.1093/geroni/igx012 30480109 PMC6177019

[51] Brooks H., L, et al, Is it time to abandon care planning in mental health services? A qualitative study exploring the views of professionals, service users and carers. Health Expectations, 2018 21: p. 597–605. 10.1111/hex.12650 29144591 PMC5980609

[52] Nicaise P., Lorant V., and Dubois V., Psychiatric advance directives as a complex and multistage intervention: a realist systematic review. Health Soc Care Comm, 2013 21: p. 1–14. 10.1111/j.1365-2524.2012.01062.x 22452515

[53] De Vleminck A., et al, Barriers and facilitators for general practitioners to engage in advance care planning: A systematic review. Scandinavian Journal of Primary Health Care, 2013 31: p. 215–226. 10.3109/02813432.2013.854590 24299046 PMC3860298

[54] Graneheim U.H. and Lundman B., Qualitative content analysis in nursing research: concepts, procedures and measures to achieve trustworthiness. Nurse education today, 2004 24(2): p. 105–112. 10.1016/j.nedt.2003.10.001 14769454

[55] Chan T.E., Peart N.S., and Chin J., Evolving legal responses to dependence on families in New Zealand and Singapore healthcare. J Med Ethics, 2014 40(12): p. 861–5. 10.1136/medethics-2012-101225 23963256

[56] Lee J.E., et al, Factors influencing attitudes toward advance directives in Korean older adults. Arch Gerontol Geriatr, 2018 **74**: p. 155–161. 10.1016/j.archger.2017.10.008 29112876

[57] Singh N., K, Zhang S., and Besmel P., Globalization and language policies of multilingual societies: Some case studies of south east Asia. Revista Brasilerira de Linguistica Aplicada, 2012 12(2): p. 349–380.

[58] Cheng H., W, B, et al, Dealing with death taboo: Discussion of do-not-resuscitate directives with Chinese patients with noncancer life-limiting illnesses. American Journal of Hospice and Palliative Medicine, 2019 36(9): p.760–766. 30744386 10.1177/1049909119828116

[59] Dunlop C. and Sorinmade O., Embedding the Mental Capacity Act 2005 in clinical practice: an audit review. Psychiatric Bulletin, 2014 38: p. 291–293. 10.1192/pb.bp.114.046870 PMC4248166 25505630

[60] Hickey D., P, C. Shriner J, and S. Perry E, Patient's initiation of advance care planning discussions with their family physician. Fam Med, 2005 **37**: p. 536 16145624

[61] Synder S., et al, Physician knowledge, attitude and experience with advance care planning, palliative care, and hospice: Results of a primary care survey. Am J Hosp Palliat Med, 2013 30(5): p. 419–424. 10.1177/1049909112452467 22798634

[62] Atkinson J., M, Garner H., C, and H. Gilmour W, Models of advance directives in mental health care: Stakeholder views. Social Psychiatry and Psychiatric Epidemiology, 2004 39: p. 673–680. 10.1007/s00127-004-0788-7 15300379

[63] de Haan L., van Raaij B, et al, Preferences for treatment during a first psychotic episode. Eur Psychiatry, 2001 16(2): p. 83–89. 10.1016/s0924-9338(01)00542-9 11311171

[64] Kennedy A., et al, Implementing a social network intervention designed to enhance and diversify support for people with long-term conditions. A qualitative study. Implement Sci, 2016 11: p. 27 10.1186/s13012-016-0384-8 26926837 PMC4772323

[65] Brooks H., Pilgrim D., and Rogers A., Innovation in mental health services: what are the key components of success? Implement Sci, 2011 6: p. 120 10.1186/1748-5908-6-120 22029930 PMC3214129

