# Supplementary material for: Stakeholder perspective on barrier to the implementation of Advance Care Planning in a traditionally paternalistic healthcare system
Source: PLoS One. 2020 Nov 10;15(11):e0242085. doi: 10.1371/journal.pone.0242085 (PMC7654826; doi:10.1371/journal.pone.0242085)
Supplement: S1 File — (DOCX) [file pone.0242085.s001.docx]

Qualitative interview guide

Service User Participants

*Advance care planning in mental health: a first qualitative phase*

1. Can you tell me a bit about yourself so we can get to know each other?
2. Can you tell me a bit about your mental illness?
3. Can you tell me about the last time you lost capacity [could not make your own decision] during an emergency or crisis? Who made the decisions for you?
4. What decisions do you think were made for you?
5. Did anyone ever offer to complete an advance care plan or advanced medical directive?
6. Can you tell me a bit about your regular treatment?
7. Is there anything you would like to add to your treatment you think might be missing?
8. Do you think the treatments you receive have been helping you? Why or why not?
   1. Is it your decision to come see doctors? Or are you obligated?
   2. Do you come to the doctor so he or she can tell you how to get better? )some people are annoyed with doctors asking them how they can help rather than telling them how they can help)
   3. Or Do you come to the doctor so he or she can help you achieve your goals?
9. Would you be willing to plan the treatment for your next crisis before you get unwell again (Planning an advance care plan)?
10. Do you think that the doctors would be able to do everything you would like them to do to help you? Why or why not?
11. How would you feel if the doctors could not do what you had planned and what they said they would do?
12. What are the situations where you would want your previous treatment decisions to change?
13. What would you want to avoid most of your regular treatment? With what should that be replaced?
14. Do you think your family would agree with the treatment decisions you make on your own?
15. Do you think your doctor would agree with the treatment decisions you make on your own?

Qualitative interview guide

Staff Participants

*Advance care planning in mental health: a first qualitative phase*

1. Can you tell me a bit about the care you provide at IMH?
2. Are you familiar with advance directives, Ulysses pacts or advance care planning? Can you tell me about them? Who should be involved?
3. Can you tell me about a case where it would have been possible or to the patient’s advantage to have an advance care plan?
4. Do you see barriers to implementing advance care planning?
5. Do you see advantages to implementing advance care planning?
6. What elements of care do you think are the most challenging?
7. Which treatment decisions do you think patients resist the most?
8. Do you think that they have alternatives to these unpopular choices?
9. When do you think you would have to override the decisions of the service users?
10. Can you think of a situation where the best interests of patients and least restrictive method of treatment was incorrectly overridden in favour of easier methods of care? Do you feel there are consequences to this?
11. How do you feel about implementing these types of tools into your daily practice?
12. In what ways do you think care could be improved?
13. In which aspects of care do you think that the physician decision and patient choice are the most incongruent?
14. What care decisions align the most (between physician decision and patient choice)?
